# The Effect of the Organizational Climate on the Integrative–Qualitative Intentional Behavior in Romanian Preschool Education—A Top-Down Perspective

**DOI:** 10.3390/bs13040342

**Published:** 2023-04-19

**Authors:** Adela Redeș, Dana Rad, Alina Roman, Mușata Bocoș, Olga Chiș, Claudiu Langa, Daniela Roman, Daniel Mara, Elena-Lucia Mara, Alina Costin, Editha Coșarbă, Ciprian Baciu

**Affiliations:** 1 Faculty of Psychology and Educational Sciences, Babeş-Bolyai University of Cluj-Napoca, 400029 Cluj-Napoca, Romania; adela_redes@yahoo.com (A.R.); musata.bocos@ubbcluj.ro (M.B.); olga.chis@ubbcluj.ro (O.C.); ciprian.baciu@ubbcluj.ro (C.B.); 2 Center of Research Development and Innovation in Psychology, Faculty of Educational Sciences Psychology and Social Work, Aurel Vlaicu University of Arad, 310032 Arad, Romania; costintalina@gmail.com (A.C.); ecosarba@yahoo.com (E.C.); 3 Department of Educational Sciences, Faculty of Education Social Sciences and Psychology, University of Pitesti, 110040 Pitesti, Romania; claudiu.langa@upit.ro; 4 Department of Psychology, Faculty of Socio-Humanistic Sciences, University of Oradea, 410087 Oradea, Romania; daniela13_roman@yahoo.com; 5 Faculty of Social Sciences and Humanities, Lucian Blaga University of Sibiu, 550024 Sibiu, Romania; daniel.mara@ulbsibiu.ro (D.M.); lucia.mara@ulbsibiu.ro (E.-L.M.)

**Keywords:** Marzano’s Model of Teaching Effectiveness, integrative–qualitative intentional behavior, serial mediation analysis, top-down perspective, Romanian preschool education, sustainable educational management

## Abstract

The concept of educational organizational climate relates to the relational, social, psychological, affective, intellectual, cultural and moral environment that characterizes educational/teaching and managerial activity at the level of a school organization. This study is based on the theory of planned behavior framework in measuring preschool teachers’ intentional integrative–qualitative behaviors and Marzano’s Model of Teaching Effectiveness. The Marzano Model outlines educational strategies and gives teachers and administrators tools to help teachers become more effective. A sample of 200 valid responses was gathered in an online investigation that targeted preschool educators from Romania. Marzano’s Model of Teaching Effectiveness is an evaluation tool used to measure the success of highly effective teachers, which is further utilized in this study to measure preschool teachers’ effectiveness in relation to intentional integrative–qualitative behaviors. The integrative–qualitative intentional behaviors are measured with the IQIB scale. This research assumes collegiality and professionalism as independent variables and interrogates preschool teachers’ behavioral intention toward adopting integrative–qualitative behaviors through the sequential mediators of Planning and Preparing, Reflecting on Teaching and Classroom Strategies and Behaviors from a top-down perspective. The results revealed a significant indirect effect of Collegiality and Professionalism on preschool teachers’ behavioral intention toward adopting intentional integrative–qualitative behaviors through the sequential mediators Planning and Preparing, Reflecting on Teaching and Classroom Strategies and Behaviors, confirming our hypothesis. Discussion and implications are offered from a top-down sustainable educational management perspective.

## 1. Introduction

A key aspect of the common teaching experience at the pre-school level is the relationships that exist between educators and preschoolers. Teachers have a significant impact on the lives of young learners. According to [1], interactions between educators and pupils are a valuable resource for and a positive influence on children’s growth. These connections can support a child’s growth by enhancing outcomes or they might create vulnerability, danger and conflict. In fact, a number of children’s outcomes are associated with teacher-preschooler interactions [2]. Particularly in preschool settings, it has been demonstrated that macro-level variables such as formal program regulations and the standards of the learning environment are less effective predictors of children’s favorable outcomes than teacher-child connections [3]. Similar to this, poor teacher-preschooler interactions have been associated with unsatisfactory behavioral and academic performance among young children as well as unfavorable viewpoints toward the educational system [4,5,6].

A school climate that characterizes the educational/teaching activity of a school micro-group/psychosocial formation has proven to be the most influential factor shaping the individual behaviors of teachers globally [7,8,9]. An educational institution’s organizational climate is the collection of internal traits that sets one school apart from another and affects how its teachers behave [10].

An alternative method of processing information and organizing knowledge is the top-down approach. According to several studies, top-down management, which exercises direct control, is still a more effective way of taming the turmoil brought on by instructors mired in unsettling and perplexing transition processes [11]. Also, it is stated that top-down management’s performance in the contemporary environment of globalization depends on the educational institution’s preparedness or desire to permit it to exist [12,13].

From the current perspective of SDG4.2, the UN Agenda specifically encourages all national governments and the international community to take steps to guarantee that all children are given equal and fair access to high-quality pre-primary education so that they are prepared for primary school [14,15,16]. Early childhood development lays the groundwork for future health, learning and well-being [17,18,19].

A research foundation that covers the critical subject of preschool teaching effectiveness is required to comprehend these crucial interconnections and encourage their positive growth and their influence on teachers’ individual behavioral intentions regarding adopting intentional integrative–qualitative behaviors. By integrative–qualitative behaviors, we mean the preschool teachers’ behavioral orientations toward an educational strategy that is both qualitative and integrative, ensuring that all preschoolers receive the same qualitative educational services [14,15].

The present study aims to investigate the impact of educational organizational climate on preschool teachers’ intentional integrative–qualitative behaviors, using the theory of planned behavior framework and Marzano’s Model of Teaching Effectiveness. The research is novel as it addresses a gap in the literature regarding the relationship between organizational climate and teachers’ adoption of integrative–qualitative behaviors in preschool settings.

Educational organizational climate is a broad concept that encompasses various aspects of the school environment, including social, psychological, affective, intellectual, cultural, and moral dimensions. Organizational climate can have a significant impact on teachers’ attitudes, beliefs, and behaviors, and ultimately affect the quality of education provided to students.

The study uses the theory of planned behavior framework to measure preschool teachers’ intentional integrative–qualitative behaviors. This theoretical framework assumes that human behavior is determined by three factors: attitude, subjective norms, and perceived behavioral control. In the context of this study, teachers’ attitudes toward adopting integrative–qualitative behaviors, their perceptions of social norms regarding such behaviors, and their perceived control over adopting them are measured.

The research also employs Marzano’s Model of Teaching Effectiveness as an evaluation tool to measure the success of highly effective teachers. The model provides educational strategies and tools to help teachers become more effective, which is particularly relevant in the context of the study. The model’s focus on effective teaching behaviors can be used to identify and measure the impact of the organizational climate on teachers’ intentional integrative–qualitative behaviors.

Adopting a top-down perspective, the present study examined the relationship between Marzano’s Model of Teaching Effectiveness investigated through the four domains (Domain 1: Classroom Strategies and Behaviors, Domain 2: Planning and Preparing, Domain 3: Reflecting on Teaching and Domain 4: Collegiality and Professionalism) and their direct and indirect impact on teachers’ individual behavioral intention toward adopting intentional integrative–qualitative behaviors.

## 2. Literature Review

The task of teaching is a difficult and complex activity, especially in early childhood environments [20,21,22,23,24]. A talented teacher employs a didactic fusion of real-world experience, discretion, enthusiasm, instructional methodologies, and flexibility to meet the various learning needs of each child [25]. Therefore, with persistent practice of research-based teaching techniques, any devoted educator may improve with time [26,27]. It may be challenging and requires some expertise and patience to control the cognitive and non-cognitive behavior of preschool children [28]. Dealing with challenging behaviors such as tired, irritable children is one of the daily challenges of educating preschoolers [29]. Not all children learn and develop at the same rate; some children could experience learning difficulties that need extra care [30]. Also, children might experience separation anxiety due to separation from their parents [31,32].

According to research, high-quality preschool is essential for preparing children for success and bridging the achievement gap [33]. The research also demonstrates that the effectiveness of preschool programs determines the advantages of early childhood education [34,35,36]. Even though a preschool educational institution may appear to have all the measurable characteristics, four essential qualities are particularly important: the preschool’s rules and procedures for handling discipline, the setting in which the child will learn, the quality of the instructors, and the resources available to them.

Educator performance standards are descriptions of theoretical knowledge and practical skills that are expected of teachers and they are becoming increasingly important in modern calls to reconsider teacher evaluation strategies. One of the most influential models for evaluating teacher effectiveness is Marzano’s Model of Teaching Effectiveness. This model is based on a thorough examination of the literature and is unique in that it has been tested through action research studies in the field [37,38].

The core foundation of this model is the belief that teaching improves through a cyclical learning-from-practice approach. The assessment of teacher effectiveness is centered around this approach. Research on the development of expert performance has shown that individuals only significantly improve through repeated practice and experience [39]. In other words, teachers become more effective by continually reflecting on their teaching practices and making improvements based on what they have learned.

By implementing performance standards and using evaluation models such as Marzano’s, educational institutions can encourage teachers to engage in reflective practice and continuous improvement, ultimately improving the quality of education for students. This approach requires a commitment to ongoing professional development and a willingness to embrace change, but it can lead to more effective teaching practices and better student outcomes.

Extensive purposeful practice combined with high-quality feedback is what results in the establishment of expert practice. By giving the learner relevant, timely, and real evidence of the caliber of his or her performance, mentors can help in the construction of purposeful practice activities that focus on crucial practice components and provide high-quality feedback. It is crucial to provide educators the time and support to reflect on their experiences since they learn more from doing so than from engaging in them [40].

There are 4 domains, 19 subdomains, and 60 indicators in the Marzano teacher assessment system [37,38]. The 60 indicators are organized over the four domains, which are: Classroom Strategies and Behaviors (Marzano’s first domain), Planning and Preparation (Marzano’s second domain), Reflection on Teaching (Marzano’s third domain), and Collegiality and Professionalism (Marzano’s fourth domain). The first domain focuses on the teacher’s classroom actions, which have an immediate effect on the academic achievement of the children. It offers the structure necessary to create a shared vocabulary throughout all groups. Moreover, it serves as a basic framework for lesson planning and as a tool for feedback and observation in the classroom. This domain requires the application of the proper strategy during the appropriate lecture section. The second domain refers to activities that are closely connected to classroom tactics and behaviors in a causal chain. For the best results in student learning, effective planning and preparation enable more intelligent judgments in the classroom. The capacity of teachers to transform their self-knowledge into professional development plans that are monitored and altered as necessary is described by the third domain, which deals with educators’ understanding of their own instructional techniques. While not being immediately tied to classroom tactics and behaviors, the fourth domain offers a framework within which the other domains may be successfully used. Collegiality and Professionalism not only describe the qualities of the school but also each teacher and administrator’s personal accountability [37,38].

These four dimensions are not independent of each other and they work together to support effective teaching and learning. For example, teachers who engage in collegial collaboration are more likely to reflect on their teaching practices and teachers who reflect on their practices are more likely to make changes to improve student outcomes. Planning and preparing instruction also depend on an understanding of effective classroom strategies and behaviors. By considering these four dimensions together, teachers can improve their teaching effectiveness and support student learning.

**Figure 1:**
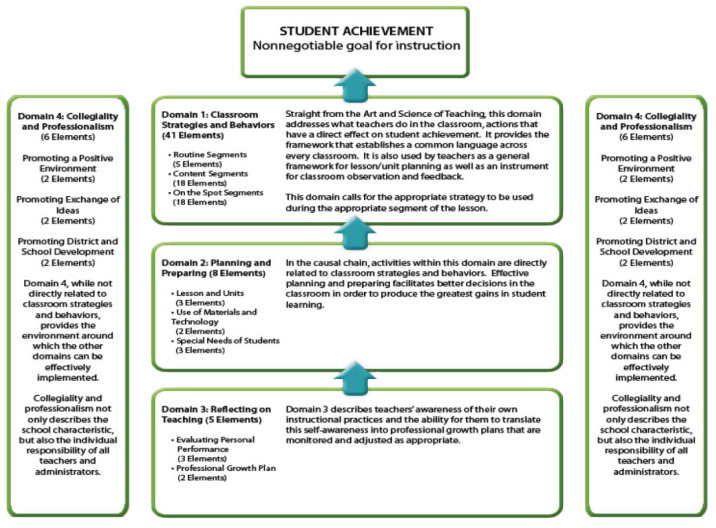
Four Domains of the Marzano Teacher Evaluation Model (Source: Marzano Center https://www.learningsciences.com/wp-content/uploads/2020/06/The-Marzano-Teacher-Evaluation-Model.pdf (accessed on 1 March 2023)).

The Marzano model’s instructions and procedures are presented in Figure 1, in order to assist teachers in self-reflection and setting development objectives. The managerial activities and procedures are presented to aid in observing the teacher and providing the educator with insightful feedback on their performance. The assessment model requests that teachers evaluate their own degrees of proficiency as a crucial first step in establishing precise professional development objectives. The goal-setting stage requires the teacher to create specific action plans and deadlines that contain the prerequisites they have determined are necessary to achieve the desired performance level for each objective. Support may come in the form of, but is not limited to: peer and mentor/coach feedback; professional development opportunities; the teacher tracking their own evolution; and chances to see and discuss the successful application of development-oriented strategies and behaviors [37,38].

Research found positive correlations between teachers’ use of the Marzano Teacher Evaluation Model and students’ learning and achievement [41,42,43,44,45,46,47].

Promoting the values and behaviors necessary for a child to function well in a classroom, get along with others, and accomplish their objectives as they mature are the purposes of managing childhood behavior and conduct [48]. Classroom management refers to all the strategies and techniques teachers employ to keep young learners interested, on track, organized, and acting politely during classes. This may include everything from body language to time management techniques, classroom management, visual resources, and digital tools. The majority of teachers create their own classroom management techniques over time and no two teachers’ methods are identical [49]. This aspect demonstrates the positive association we might expect between the first and second Marzano domains: classroom strategies and behaviors (Marzano’s first domain), and planning and preparing (Marzano’s second domain).

The daily, monthly, or quarterly preparation that a teacher could conduct to be ready for classes or activities is included in the planning strategy. This can include any reflexive procedures a teacher employs to be ready to teach a lesson, anticipated through the lesson design’s approach. Before, during, and after a class is delivered, reflective teaching entails assessing one’s fundamental assumptions about teaching and learning as well as one’s alignment with actual classroom practice. By teaching reflectively, teachers examine their methods critically and seek out examples of good instruction [50,51,52,53,54,55,56]. This aspect demonstrates the positive association we might expect between the second and third Marzano domains, between planning and preparing (Marzano’s second domain) and reflecting on teaching (Marzano’s third domain).

In order to guarantee that their learning has an influence on their practice and professional development, [57] offers teachers a framework within which to design, participate in, and reflect on their own learning. It is accepted that there is no “one size fits all” approach to reflection and educators are encouraged to build strategies that suit their unique learning styles and institutional and instructional situations [58,59,60,61]. At all levels of the educational system, creating a culture of professional development among teachers is becoming increasingly important. It is still required to research teachers’ shared reflective experiences in order to close the knowledge gap between the conceptualization of professional learning communities and how these learning communities are implemented and sustained in early educational environments [62,63]. These findings suggest that we can expect a positive correlation between the first and second Marzano domains, between third and fourth Marzano domains, namely reflecting on teaching (Marzano’s third domain), and collegiality and professionalism (Marzano’s fourth domain).

According to the theory of planned behavior [64], individuals respond logically in accordance with their beliefs, views, arbitrary opinions, own standards, and apparent behavioral control. Although not often actively or consciously taken into account, these elements serve as the framework for making decisions. The need for both qualitative and integrative early childhood education has further been investigated in this study based on the theory of planned behavior.

Regarding the interconnection of all four Marzano domains and the need for both qualitative and integrative early childhood education, the objective of this study is to analyze from the perspective of the Marzano-type evaluation how the organizational climate in preschool educational institutions in Romania, influences the integrative–qualitative intentional behavior of preschool educators. More specifically, we intend a serial or sequential full complementary mediation of Planning and Preparing, Reflecting on Teaching and Classroom Strategies and Behaviors over the relationship between Collegiality and Professionalism and preschool teachers’ behavioral intention toward adopting integrative–qualitative behaviors (IQIB).

## 3. Materials and Methods

### 3.1. Research Design

Sequential mediation is a statistical model used in social science research to investigate the indirect effects of an independent variable on a dependent variable through a series of mediators. It assumes that the relationship between independent variables and dependent variables is partially or fully mediated by one or more mediators and that the mediators themselves are interdependent, with one mediator affecting the next in a causal chain. In a sequential mediation model, the effect of the independent variable on the dependent variable is broken down into a series of direct and indirect effects through multiple mediators. The model allows for the testing of whether the indirect effect of the independent variable on the dependent variable through a first mediator is further mediated by a second mediator. This can help researchers understand the underlying mechanisms of how an intervention or treatment affects the outcome of interest. To estimate the parameters of a sequential mediation model, researchers typically use structural equation modeling (SEM) or path analysis. This involves estimating a series of regression equations to model the relationships between the independent variables, mediators, and dependent variables. The model can then be tested for its goodness-of-fit to the data and the significance of indirect effects. Sequential mediation is a useful tool for researchers who want to better understand the complex causal pathways through which their independent variables affects their dependent variables. By identifying and testing the mediators that link the two variables, researchers can gain insights into the specific mechanisms through which an intervention or treatment works and potentially identify new targets for intervention.

Following the analysis of the descriptive statistics for each research variable and establishing the scales’ reliability coefficients, we investigated, using a sequential mediation analysis, the effect of Marzano’s teacher effectiveness domains over preschool teachers’ behavioral intention toward adopting integrative–qualitative behaviors. For statistical analysis purposes, we have used SPSS Process Macro model 6, which was designed for testing serial or sequential mediations with up to 4 mediators [65].

Based on the literature review, we tested the sequential mediation effect of Planning and Preparing, Reflecting on Teaching, and Classroom Strategies and Behaviors on the relationship between Collegiality and Professionalism and preschool teachers’ behavioral intention toward adopting integrative–qualitative behaviors (IQIB).

By testing the sequential mediation effect, we were able to explore whether the four domains of the Marzano model acted as a chain of mediators, sequentially transmitting the effect of Collegiality and Professionalism on preschool teachers’ behavioral intention toward adopting IQIB. Specifically, we investigated the hypothesis that Collegiality and Professionalism would predict Planning and Preparing, which in turn would predict Reflecting on Teaching, which would finally predict Classroom Strategies and Behaviors, leading to the ultimate outcome of preschool teachers’ behavioral intention toward adopting IQIB. By examining the sequential mediation effect, we will be able to gain insights into the specific ways in which the different domains of the Marzano model are related to preschool teachers’ behavioral intention toward adopting IQIB and the specific paths through which these relationships operated. This can help to inform the development of targeted interventions and strategies to improve teacher effectiveness and promote the adoption of effective teaching practices.

The hypothesis of this research states that Planning and Preparing, Reflecting on Teaching, and Classroom Strategies and Behaviors represent three sequential mediators on the relationship between Collegiality and Professionalism and preschool teachers’ behavioral intention toward adopting integrative–qualitative behaviors (IQIB). Using SPSS V.26 Process Model 6, which assumes the detection of a serial mediation effect—that is, a causal chain connecting three mediators—we evaluated this hypothesis. Process model 6 is also referred to as sequential mediation analysis.

### 3.2. Participants

Preschool educators were enlisted for this study, which involved a survey utilizing a convenience sample technique. The online survey was widely disseminated across social media groups for preschool instructors in the west of Romania. In all, 224 valid and confirmed replies were received throughout the months of October and November 2022. The ages of the respondents ranged from 20 to 64 with a mean age of 36. All 224 responders were female preschool teachers, which represent a gender nondiscriminatory sample due to the fact that in Romania, fewer than 5% of all preschool teachers are male.

The respondents’ previous teaching experience spanned from 1 to 44 years with a mean of 12 years of previous preschool teaching experience. Regarding the educational institutions where they work, 32% of the preschool educational institutions are rural and 68% are in an urban environment. In addition, 82% of the institutions are public preschool educational institutions and 18% are private preschool educational institutions from West Romania.

In terms of professional status, 3% are working as unqualified substitute preschool teachers, 21% are qualified substitute preschool teachers, and 76% are permanent preschool teachers. All percentages are calculated based on the individuals. Regarding the respondents’ professional degrees, 49% obtained a final certificate in preschool education, 38% have an education degree (level 2) or advanced teaching skills, and 13% have an education degree (level 1) or the highest-level teaching proficiency qualification under Romanian regulations.

Each respondent voluntarily consented to take part in the study and granted permission for the release of their replies’ aggregated data.

### 3.3. Research Instruments

The instruments used in this research are the Marzano Teacher Evaluation Model Instrument developed by [57,58], and the Integrative–qualitative Intentional Behavior in Preschool Education Scale (IQIB-ECEC) developed by [14].

The Marzano Teacher Evaluation Model Instrument was adapted for the Romanian preschool context in the Romanian language. In this particular study, the questionnaires, after being adapted for preschool education, were made available in Romanian, the language spoken in the research context. To ensure the reliability and validity of the instrument in Romanian, several steps were taken. First, the original English version of the questionnaires were translated into Romanian by professional translators who were fluent in both languages. The translated version was then back-translated into English by a second group of translators who were also fluent in both languages. This step was taken to ensure that the original meaning of the English version was preserved in the Romanian translation. After the translation process was completed, the instrument was reviewed by a panel of experts in the field who were also fluent in both languages. This review aimed to ensure that the questions were culturally appropriate and relevant to the research context. Once the review was completed, the instrument was then pre-tested with a group of Romanian teachers. This pre-test aimed to identify any issues with the questions, such as confusing or unclear wording, that may impact the validity of the instrument. Based on the feedback received from the pre-test, the instrument was revised and finalized in Romanian. This final version of the instrument was then used to collect data from the participants in the study. Overall, these steps were taken to ensure that the questionnaires were reliable and valid in the Romanian language and that they accurately captured the Marzano model.

The Marzano model consists of 60 indicators divided into four domains of teacher effectiveness assessment. The four domains assessed are Classroom Strategies and Behaviors, Planning and Preparing, Reflecting on Teaching, and Collegiality and Professionalism. Each domain has several sub-domains and the 60 indicators are balanced among the domains. The first domain focuses on teachers’ classroom activities, which have a direct impact on students’ academic performance. The second domain is closely connected to classroom strategies and behaviors. The third domain deals with instructors’ understanding of their own instructional techniques and their capacity to transform their self-knowledge into professional growth plans. The fourth domain provides a framework within which the other domains may be successfully used, focusing on collegiality and professionalism as qualities of the school and the personal accountability of each teacher.

The preschool teachers rated all items on a 5-point Likert-type scale, with 1 indicating strong disagreement, 2 indicating disagreement, 3 indicating neutral, 4 indicating agreement, and 5 indicating strong agreement. The scores for all four domains were obtained by averaging the 60 corresponding indicators for the 19 sub-domains. Overall, the Marzano Teacher Evaluation Model Instrument is a comprehensive tool that assesses various domains of teacher effectiveness, including classroom strategies and behaviors, planning and preparing, reflecting on teaching, and collegiality and professionalism. The use of this instrument in the preschool context is particularly relevant given the importance of early childhood education and the need for effective teaching strategies to promote positive academic outcomes.

The IQIB-ECEC scale, on the other hand, measures intentional integrative–qualitative behaviors in preschool education. The combination of these two instruments allowed the researchers to examine the relationship between teacher effectiveness and intentional integrative–qualitative behaviors in the preschool context.

The Integrative–qualitative Intentional Behavior in Preschool Education Scale (IQIB-ECEC) developed by [14] and based on the theory of planned behavior has 19 items, referring to 8 factors: actual behavior, attitudes toward the behavior, behavioral beliefs, subjective norm, normative beliefs, perceived power/control beliefs, perceived behavioral control, and behavioral intention, with all items referring to preschool teachers’ behavioral intention toward adopting integrative–qualitative behaviors. All items were rated by the preschool teachers on a 5-point Likert-type scale with 1 = strong disagreement, 2 = disagreement, 3 = neutral, 4 = agreement, and 5 = strong agreement, referring to their own perceived behavioral intention toward adopting integrative–qualitative behaviors. All 19 items’ scores were averaged and the total score was used for statistical processing. The integrative–qualitative intentional behaviors scale reported a Cronbach’s α of 0.793 with a grand mean 4.63 of and a variance of 0.102.

## 4. Results

### 4.1. Preliminary Investigation

Before running the correlation analysis, we first checked the reliability coefficients of both measurements used in this research, using the JASP software version 0.16.3.0. Results are presented in Table 1.

The table shows the reliability statistics for the Marzano individual items and the IQIB individual items. The reliability of each item is measured using Cronbach’s α, which is a measure of internal consistency. A high value of Cronbach’s α indicates that the items are strongly correlated and the scale is reliable. For the Marzano individual items, all items have a Cronbach’s α above 0.93, indicating a high level of internal consistency. This suggests that the Marzano scale is reliable in measuring the concept it is intended to measure. For the IQIB individual items, all items except DQ16 have a Cronbach’s α above 0.77, which is considered acceptable. Only DQ16 has a relatively lower Cronbach’s α of 0.794. In conclusion, the table suggests that the both the Marzano scale and the IQIB scale have strong reliability.

The integrative–qualitative intentional behaviors scale reported a Cronbach’s α of 0.793 with a grand mean 4.63 of and a variance of 0.102. The 19 subdomains of Marzano’s Model of Teaching Effectiveness scale obtained a Cronbach’s α of 0.938 with a grand mean of 4.80 and a variance of 0.005. Both scales thus indicate strong reliability characteristics.

Subsequently, we have analyzed the correlation coefficients between the 4 domains of Marzano’s scale: Domain 1—Classroom Strategies and Behaviors by averaging the sub-dimensions DQ1 to DQ9, Domain 2 —Planning and Preparing by averaging the sub-dimensions DQ10 to DQ14, Domain 3—Reflecting on Teaching by averaging the sub-dimensions DQ15 and DQ16, Domain 4—Collegiality and Professionalism by averaging the sub-dimensions DQ17 to DQ19, and the integrative–qualitative intentional behavior scale as the overall score. Results are presented in Table 2.

As depicted in Table 3, we have observed significant correlations between all four Marzano domains and the overall IQIB score reflecting preschool teachers’ behavioral intention toward adopting integrative–qualitative behaviors (IQIB). Positive correlations spanned from 0.397 to 0.744, all being significant at *p* < 0.001.

### 4.2. Sequential Mediation Analysis Results

Subsequently, we have tested our mediation analysis using SPSS Process Macro model 6. Our serial mediation model had as dependent variable preschool teachers’ behavioral intention toward adopting integrative–qualitative behaviors (IQIB), as independent variable Collegiality and Professionalism (Marzano’s fourth domain) (D4), and as three mediators Planning and Preparing (Marzano’s second domain) (D2), Reflecting on Teaching (Marzano’s third domain) (D3) and Classroom Strategies and Behaviors (Marzano’s first domain) (D1). Based on the theory of planned behavior [64] and prior research, the three serial mediators predict the improvement of the dependent variable, the behavioral intention of preschool educators to adopt integrative–qualitative behaviors.

The mediation analysis results revealed a significant indirect effect of Collegiality and Professionalism (Marzano’s fourth domain) (D4) on preschool teachers’ behavioral intention toward adopting integrative–qualitative behaviors (IQIB) through the sequential mediators Planning and Preparing (Marzano’s second domain) (D2), Reflecting on Teaching (Marzano’s third domain) (D3) and Classroom Strategies and Behaviors (Marzano’s first domain) (D1). In the presence of the mediators, the direct effect of D4 on IQIB was no longer significant (b = −0.0375, t = −0.4372, *p* > 0.05). Hence, there is full complementary sequential mediation of D2, D3 and D1 resulting from the relationship between D4 and preschool teachers’ behavioral intention toward adopting integrative–qualitative behaviors (IQIB). Mediation summary is presented in Table 3.

The sequential mediation model predicted an overall 28% increase of preschool teachers’ behavioral intention toward adopting integrative–qualitative behaviors (IQIB), an outcome with an extremely high percentage. Marzano’s Model of Teaching Effectiveness and research results that have been published in the scientific literature confirm this finding in reference to the relationship between Collegiality and Professionalism and teachers adopting new behaviors, such as integrative–qualitative behaviors (IQIB).

Overall, the three mediators fully and complementarily mediated the relationship between Collegiality and Professionalism (D4) and preschool teachers’ behavioral intention toward adopting integrative–qualitative behaviors (IQIB), (IE = 0.0224, 95% CI: LL = 0.0023 to UL = 0.0528), indicating that preschool teachers with a high perception of Collegiality and Professionalism, Planning and Preparing, Reflecting on Teaching, and Classroom Strategies and Behaviors were more likely to have higher levels of behavioral intention toward adopting integrative–qualitative behaviors (IQIB).

## 5. Discussion

The study assumes that Collegiality and Professionalism as an independent variable impacts preschool teachers’ behavioral intention toward adopting integrative–qualitative behaviors through the sequential mediators Planning and Preparing, Reflecting on Teaching, and Classroom Strategies and Behaviors when adopting a top-down perspective.

The results of the study reveal a significant indirect effect of Collegiality and Professionalism on preschool teachers’ behavioral intention toward adopting intentional integrative–qualitative behaviors through the sequential mediators Planning and Preparing, Reflecting on Teaching, and Classroom Strategies and Behaviors, confirming the hypothesis. The findings highlight the importance of a positive organizational climate that fosters Collegiality and Professionalism in promoting teachers’ adoption of integrative–qualitative behaviors.

The study’s implications are significant from a top-down sustainable educational management perspective. The findings suggest that educational leaders should prioritize creating a positive organizational climate that promotes collegiality and professionalism to support teachers’ adoption of integrative–qualitative behaviors. This can be achieved through training, professional development, and support for teachers to enhance their planning, preparation, and reflection skills.

Overall, the study is significant as it contributes to the literature on the relationship between the organizational climate and teachers’ adoption of integrative–qualitative behaviors in preschool settings. The study’s novelty lies in its theoretical framework, the use of Marzano’s Model of Teaching Effectiveness, and the focus on a specific aspect of the organizational climate, namely, collegiality and professionalism. The findings have important implications for educational leaders, policymakers, and practitioners in promoting effective teaching practices in preschool settings.

Overall, the three mediators fully and complementarily mediated the relationship between collegiality and professionalism (D4) and preschool teachers’ behavioral intention toward adopting integrative–qualitative behaviors (IQIB), showing that preschool teachers with high perceptions of collegiality and professionalism (D4), planning and preparation (D2), reflecting on teaching (D3), and classroom strategies and behaviors (D1) were more likely to have higher levels of behavioral intention toward adopting integrative–qualitative behaviors (IQIB).

According to several studies, peer relationships and other preschool components are important factors in how children behave [66]. The interaction between children and their preschool teachers is just one of the many learning components of the classroom setting. The classroom setting design communicates the teacher’s expectations for young learners’ motivation as a premise for successful learning, the teaching methods that should be used, the kinds of resources that should be used to engage children in learning, and appropriate classroom conduct. Thus, the environment and by implication the preschool organizational culture have a direct impact on children’ motivation, social connection, and interest in the learning process. The attractiveness that is represented in the classroom and the effectiveness of the teacher’s preparation and instruction may all be regarded as indicators of a positive psychosocial and curricular classroom environment [67].

Research on collegiality’s impact on individual behaviors found that assessments of collegiality are associated with higher budgets, performance evaluation processes, and workload allocations [68]. In addition, research on the relationship between educational institution climate and teachers’ commitment revealed that poor leadership and institutional vulnerability are predictors of teachers’ decreased commitment [69]. In light of these findings, our research also identifies the main factor in enhancing preschool teachers’ levels of behavioral intention toward adopting integrative–qualitative behaviors as being organizational climate, namely perception of collegiality and professionalism.

The first complementary mediation effect is provided by planning and preparing, referring to activities that are closely connected to classroom stragegies and behaviors. The most effective method for guaranteeing that children are learning effectively, that they are moving forward as needed toward the early learning goals, and that learning is exciting and progressive is through planning [70,71]. Planning also ensures that all children have access to equal developmental opportunities and benefit from a balanced curriculum.

The second complementary mediation effect is provided by reflecting on teaching. The capacity of teachers to transform their self-knowledge into professional growth plans that are monitored and altered as necessary is described by Marzano’s third domain, which deals with instructors’ understanding of their own instructional techniques. Reflection is a high-impact technique that enhances the learning of all teachers, especially preschool teachers [72]. Preschool teachers must constantly reflect on their own beliefs, prejudices, and behaviors. By encouraging critical reflection on behaviors in a real-world setting, reflective practice helps preschool teachers build new knowledge, skills, and attitudes needed in the process of integrating and focusing on behavioral intention toward adopting integrative–qualitative behaviors [73,74,75].

The third complementary mediation effect is provided by classroom strategies and behaviors. As seen by Marzano, this domain serves as a basic framework for lesson planning and a tool for feedback and observation in the classroom. This domain requires the application of the proper strategy during the appropriate lecture section. Classroom strategies and behaviors preschool teachers adopt influence to a great extent the behavioral intention toward adopting a multitude of work related behaviors [76], this domain being the last sequential mediator that indirectly complementarily impacts behavioral intention toward adopting integrative–qualitative behaviors.

**Figure 2:**
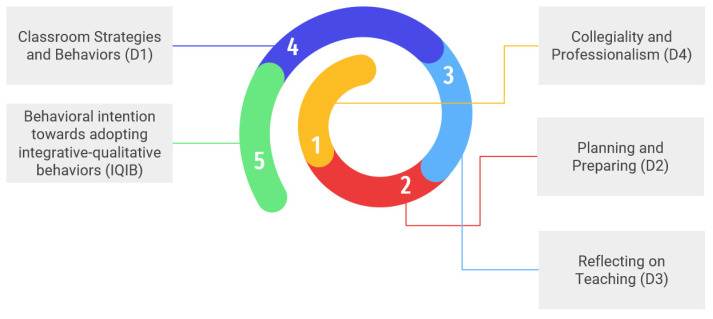
The complementary sequential mediation pathway for enhancing behavioral intention toward adopting integrative–qualitative behaviors (IQIB).

For a better visualization of our results, we propose the visual depicted in Figure 2 of the complementary sequential mediation pathway for enhancing behavioral intention toward adopting integrative–qualitative behaviors.

In Figure 2, it is depicted how preschool organizational culture, namely Marzano’s domain 4 Collegiality and Professionalism, impacts preschool teachers’ behavioral intention toward adopting integrative–qualitative behaviors firstly through Marzano’s domain 2 Planning and Preparing, then through domain 3 Reflecting on Teaching and lastly through domain 1 Classroom Strategies and Behaviors, clearly depicting the top-down perspective, from preschool organizational culture toward the individual behavior of adopting integrative–qualitative behaviors.

Preschool education is a crucial stage of learning where children develop social, cognitive, and emotional skills that form the foundation for their future academic and personal success. The role of preschool teachers is essential in ensuring quality of education and fostering positive developmental outcomes for young learners. Effective preschool teaching requires intentional integrative–qualitative behavior, which involves integrating a range of teaching strategies and techniques to meet the diverse needs of learners. The Marzano model is an evidence-based framework for effective teaching and learning that can guide school management and educational policies to influence preschool teachers’ intentional integrative–qualitative behavior and learner outcomes. In this discussion, we will examine how the Marzano model can be applied to preschool education and provide practical advice to school management and policymakers to leverage changes in preschool teachers’ behavior and learner outcomes.

Practical Advice for School Management and Educational Policies:

- Provide Professional Development Opportunities: School management and educational policies can provide professional development opportunities for preschool teachers to enhance their knowledge and skills. Professional development programs can focus on the Marzano model’s key components, such as classroom management, instructional strategies, and assessment practices. This can help teachers develop intentional integrative qualitative behavior and improve learner outcomes.
- Foster Collaborative Learning Communities: Preschool teachers can benefit from a collaborative learning community where they can share their experiences, ideas, and strategies. School management and educational policies can support the creation of such communities and provide resources to facilitate collaborative learning. This can help teachers develop intentional integrative qualitative behavior and improve learner outcomes.
- Use Data to Inform Instruction: School management and educational policies can encourage the use of data to inform instructional decisions. Teachers can use data to assess student learning and adjust their teaching strategies accordingly. The Marzano model emphasizes the importance of data-driven instruction and can guide teachers to use data effectively to improve learner outcomes.
- Provide a Safe and Supportive Learning Environment: School management and educational policies can provide a safe and supportive learning environment for preschool learners. A positive and nurturing environment can promote social and emotional development and foster positive learner outcomes. The Marzano model emphasizes the importance of a positive classroom environment and provides strategies to create such an environment.
- Emphasize Student-Centered Learning: School management and educational policies can emphasize student-centered learning in preschool education. Learners are more engaged and motivated when they have a sense of ownership over their learning. The Marzano model emphasizes the importance of student-centered learning and provides strategies to create such an environment.

## 6. Conclusions

Assessments of preschool teacher efficacy are worldwide used to enhance preschool educational services. The ultimate purpose of assessment is to enhance education outcomes and children’s learning and there are several methods of quantifying teacher effectiveness and numerous applications for evaluation outcomes. This research is based on the theory of planned behavior framework in measuring preschool teachers’ intentional integrative–qualitative behaviors and Marzano’s Model of Teaching Effectiveness.

The Marzano Model outlines educational strategies and gives teachers and administrators tools to help teachers become more effective. A sample of 200 valid responses was gathered in an online investigation that targeted preschool educators from West Romania. Marzano’s Model of Teaching Effectiveness is an evaluation tool used to measure the success of highly effective teachers, which is further utilized in this research to measure preschool teachers’ effectiveness in relation to intentional integrative–qualitative behaviors. The intentional integrative–qualitative behaviors are measured with IQIB scale.

In accordance with the literature review, this study finds that Collegiality and Professionalism (Marzano fourth domain) as independent variable impacts preschool teachers’ behavioral intention toward adopting integrative–qualitative behaviors through the sequential mediators Planning and Preparing (Marzano’s second domain), Reflecting on Teaching (Marzano’s third domain) and Classroom Strategies and Behaviors (Marzano’s first domain), when adopting a top-down perspective.

After the preliminary investigation that was focused on assessing the reliability of the scales, a sequential mediation analysis was computed in SPSS V.26 with Process Macro number 6. The results revealed a significant indirect effect of Collegiality and Professionalism on preschool teachers’ behavioral intention toward adopting intentional integrative–qualitative behaviors through the sequential mediators Planning and Preparing, Reflecting on Teaching, and Classroom Strategies and Behaviors, supporting our hypothesis. Furthermore, the direct effect of Collegiality and Professionalism on preschool teachers’ behavioral intention toward adopting intentional integrative–qualitative behaviors in the presence of the mediators was no longer found significant. Hence, there is a full complementary sequential mediation of Classroom Strategies and Behaviors (Marzano’s first domain) and Planning and Preparing (Marzano 2nd domain) on the relationship between collegiality and professionalism and behavioral intention toward adopting integrative–qualitative behaviors.

Preschool education plays a crucial role in shaping learners’ social, emotional, and cognitive development. Effective preschool teaching requires intentional integrative–qualitative behavior, which involves integrating a range of teaching strategies and techniques to meet the diverse needs of learners. The Marzano model provides an evidence-based framework for effective teaching and learning that can guide school management and educational policies to influence preschool teachers’ behavior and learner outcomes. By providing professional development opportunities, fostering collaborative learning communities, using data to inform instruction, providing a safe and supportive learning environment, and emphasizing student-centered learning, school management and educational policies can leverage changes in preschool teachers’ behavior and learner outcomes.

## Figures and Tables

**Table 1:** Reliability statistics of the Marzano and IQIB scales.

Marzano Individual Item Reliability Statistics					IQIB Individual Item Reliability Statistics				
Item	Domain	If Item Dropped Cronbach’s α	Mean	SD	Item	Domain	If Item Dropped Cronbach’s α	Mean	SD
DQ1	Domain 1 Classroom Strategies and Behaviors	0.935	4.853	0.331	DQ1	D1. Actual behavior	0.787	4.866	0.379
DQ2		0.936	4.868	0.310	DQ2		0.788	4.862	0.371
DQ3		0.933	4.830	0.328	DQ3	D2. Attitudes	0.790	4.902	0.313
DQ4		0.932	4.801	0.386	DQ4		0.786	4.853	0.435
DQ5		0.935	4.728	0.497	DQ5	D3. Behavioral beliefs	0.793	4.960	0.218
DQ6		0.933	4.827	0.328	DQ6		0.794	4.991	0.094
DQ7		0.934	4.853	0.348	DQ7	D4. Subjective norm	0.792	4.647	0.625
DQ8		0.934	4.874	0.329	DQ8		0.781	4.576	0.602
DQ9		0.942	4.661	0.646	DQ9	D5. Normative beliefs	0.789	4.777	0.579
DQ10	Domain 2 Planning and Preparing	0.934	4.871	0.307	DQ10		0.787	4.576	0.777
DQ11		0.935	4.868	0.350	DQ11		0.791	4.576	0.723
DQ12		0.934	4.879	0.365	DQ12	D6. Control beliefs	0.780	4.335	0.757
DQ13		0.942	4.661	0.722	DQ13		0.774	4.656	0.616
DQ14		0.936	4.737	0.582	DQ14		0.774	4.366	0.781
DQ15	Domain 3 Reflecting on Teaching	0.931	4.804	0.422	DQ15	D7. Perceived behavioral control	0.777	4.478	0.825
DQ16		0.937	4.699	0.595	DQ16		0.794	3.598	1.238
DQ17	Domain 4 Collegiality and Professionalism	0.933	4.864	0.342	DQ17	D8. Behavioral intention	0.766	4.469	0.775
DQ18		0.934	4.815	0.413	DQ18		0.776	4.799	0.492
DQ19		0.934	4.804	0.423	DQ19		0.776	4.790	0.524

**Table 2:** Pearson’s Correlations.

Variable	Domain 1		Domain 2		Domain 3		Domain 4		IQIB
1. Domain 1—Classroom Strategies and Behaviors	—								
2. Domain—Planning and Preparing	0.724	***	—						
3. Domain—Reflecting on Teaching	0.686	***	0.707	***	—				
4. Domain—Collegiality and Professionalism	0.765	***	0.744	***	0.717	***	—		
5. IQIB	0.533	***	0.397	***	0.410	***	0.408	***	—

*** *p* < 0.001.

**Table 3:** Sequential mediation results.

Total Effect (D4 → IQIB)	Direct Effect (D4 → IQIB)	Relationship	Indirect Effect	Confidence Intervals		t = Indirect Effect/SE	Conclusion
				Lower Bound	Upper Bound		
0.3426 *p* < 0.0001	−0.0375 *p* > 0.05	H1: D4 → D2 → D3 → D1 → IQIB	0.0224	0.0023	0.0528	1.7230	Full complementary mediation

## Data Availability

The authors will make the raw data supporting the conclusion of this study available without restriction.
